# RETRACTION: Effect of Minimally Invasive Surgery and Laparotomy on Wound Infection and Postoperative and Intraoperative Complications in the Management of Cervical Cancer: A Meta‐Analysis

**DOI:** 10.1111/iwj.70467

**Published:** 2025-04-02

## Abstract

**RETRACTION:** ZhengS. , LiuX. , ChengL. , WuQ. , and MengF. , “Effect of Minimally Invasive Surgery and Laparotomy on Wound Infection and Postoperative and Intraoperative Complications in the Management of Cervical Cancer: A Meta‐Analysis,” International Wound Journal 20, no. 4 (2023): 1061–1071, 10.1111/iwj.13962.36111540 PMC10031228

The above article, published online on 16 September 2022, in Wiley Online Library (http://onlinelibrary.wiley.com/), has been retracted by agreement between the journal Editor in Chief, Professor Keith Harding; and John Wiley & Sons Ltd. Following an investigation by the publisher, all parties have concluded that this article was accepted solely on the basis of a compromised peer review process. The editors have therefore decided to retract the article. The authors did not respond to our notice regarding the retraction.

**RETRACTION:** S. Zheng , X. Liu , L. Cheng , Q. Wu , and F. Meng , “Effect of Minimally Invasive Surgery and Laparotomy on Wound Infection and Postoperative and Intraoperative Complications in the Management of Cervical Cancer: A Meta‐Analysis,” International Wound Journal 20, no. 4 (2023): 1061–1071, 10.1111/iwj.13962.36111540 PMC10031228

The above article, published online on 16 September 2022, in Wiley Online Library (http://onlinelibrary.wiley.com/), has been retracted by agreement between the journal Editor in Chief, Professor Keith Harding; and John Wiley & Sons Ltd. Following an investigation by the publisher, all parties have concluded that this article was accepted solely on the basis of a compromised peer review process. The editors have therefore decided to retract the article. The authors did not respond to our notice regarding the retraction.

